# Automated Production of [^68^Ga]Ga-Desferrioxamine B on Two Different Synthesis Platforms

**DOI:** 10.3390/pharmaceutics16091231

**Published:** 2024-09-21

**Authors:** Martin Kraihammer, Miloš Petřík, Christine Rangger, Michael Gabriel, Hubertus Haas, Bernhard Nilica, Irene Virgolini, Clemens Decristoforo

**Affiliations:** 1 Department of Nuclear Medicine, Medical University Innsbruck, Anichstrasse 35, A-6020 Innsbruck, Austria; martin.kraihammer@i-med.ac.at (M.K.); christine.rangger@i-med.ac.at (C.R.); bernhard.nilica@i-med.ac.at (B.N.); irene.virgolini@i-med.ac.at (I.V.); 2 Institute of Nuclear Medicine and Endocrinology, Kepler University Hospital, Krankenhausstrasse 9, A-4021 Linz, Austria; michael.gabriel@kepleruniklinikum.at; 3 Medical Faculty, Johannes Kepler University Linz, Altenberger Strasse 69, A-4040 Linz, Austria; 4 Institute of Molecular and Translational Medicine, Faculty of Medicine and Dentistry, Palacky University, CZ-77900 Olomouc, Czech Republic; milos.petrik@upol.cz; 5 Institute of Molecular Biology, Biocenter, Medical University of Innsbruck, A-6020 Innsbruck, Austria; hubertus.haas@i-med.ac.at

**Keywords:** desferrioxamine B, gallium-68, PET, infection, imaging, validation

## Abstract

**Background/Objectives:** PET imaging of bacterial infection could potentially provide added benefits for patient care through non-invasive means. [^68^Ga]Ga-desferrioxamine B—a radiolabelled siderophore—shows specific uptake by human-pathogenic bacteria like *Staphylococcus aureus* or *Pseudomonas aeruginosa* and sufficient serum stability for clinical application. In this report, we present data for automated production of [^68^Ga]Ga-desferrioxamine B on two different cassette-based synthesis modules (Modular-Lab PharmTracer and GRP 3V) utilising commercially obtainable cassettes together with a licensed ^68^Ge/^68^Ga radionuclide generator. **Methods:** Quality control, including the determination of radiochemical purity, as well as a system suitability test, was set up via RP-HPLC on a C18 column. The two described production processes use an acetic acid/acetate buffer system with ascorbic acid as a radical scavenger for radiolabelling, yielding ready-to-use formulations with sufficient activity yield. **Results:** Batch data analysis demonstrated radiochemical purity of >95% by RP-HPLC combined with ITLC and excellent stability up to 2 h after synthesis. Specifications for routine production were set up and validated with four masterbatches for each synthesis module. **Conclusions:** Based on this study, an academic clinical trial for imaging of bacterial infection was initiated. Both described synthesis methods enable automated production of [^68^Ga]Ga-desferrioxamine B in-house with high reproducibility for clinical application.

## 1. Introduction

Nosocomial bacterial infections are associated with high mortality, especially with a growing number of drug-resistant pathogens seen in routine clinical practice. Described as part of the group of ESKAPE pathogens, *Staphylococcus aureus* and *Pseudomonas aeruginosa* are the cause of many hospital-acquired infections with potential life-threatening progress [1,2]. Early diagnosis and accurate localisation of these infections could improve patient outcomes and would allow us to monitor antibacterial treatment more quickly and precisely than current diagnostic techniques. Established methods include the identification of bacteria through culture tests, which suffer from a long delay between the sampling and obtaining of results [3], or molecular imaging techniques like [^18^F]FDG, among others, which rely on the secondary inflammatory response of the host organism [4,5]. Novel radiopharmaceuticals for fast and specific detection of human pathogens could, therefore, provide a considerable benefit to clinical practice [6].

Desferrioxamine B (deferoxamine, DFO-B), a naturally occurring substance produced by *Streptomyces pilosus*, belongs to a group of biomolecules called siderophores, which show high binding affinity for iron, among other metals [7]. These low-molecular-weight (<1 kDa) structures play a pivotal role in the iron metabolism of most fungal and bacterial species, including several human pathogenic microorganisms [8]. The uptake of these siderophores involves highly specific transporter proteins, which are not found in human cells [9]. Trivalent gallium, as an isosteric diamagnetic substitute for ferric iron, also forms stable complexes with siderophores [10], facilitating targeted uptake into microbial cells. Labelling siderophores with positron-emitting Ga-68 could enable the precise localisation of pathogens by means of Positron Emission Tomography (PET), a concept already proven in preclinical imaging studies of *S. aureus* and *P. aeruginosa* mouse infection models with [^68^Ga]Ga-desferrioxamine B [11]. Data from preclinical imaging of *Aspergillus fumigatus* in a rat aspergillosis model suggest that this concept might also be transferable to fungal infections [12].

Automation of synthesis and radiolabelling procedures for bringing novel radiopharmaceuticals into clinical settings has become increasingly important in recent years. A driving force of this development is the rising use of PET radionuclides like Ga-68, which is readily available on-site from ^68^Ge/^68^Ga radionuclide generators or cyclotron production [13]. The high decay energy of such radioisotopes imposes a substantial radiation burden on the personnel performing radiopharmaceutical production, which can be reduced significantly through the use of automated synthesis modules [14] while also increasing repeatability and providing complete documentation of the production process [15].

This study aimed to establish automated production of [^68^Ga]Ga-desferrioxamine B on two different synthesis modules for clinical trial application according to current scientific standards [16,17]. Specifications for routine production of [^68^Ga]Ga-desferrioxamine B were set up based on existing monographs of the European Pharmacopeia (Ph. Eur.) for other Ga-68 labelled radiopharmaceuticals [18,19].

## 2. Materials and Methods

### 2.1. Materials and Automated Synthesis

Automated syntheses were conducted on two different automated modules using similar synthesis approaches. Method 1: Modular-Lab PharmTracer^®^ (Eckert & Ziegler Radiopharma GmbH, Berlin, Germany) in conjunction with C4-GA-PEP cassettes (Eckert & Ziegler Radiopharma GmbH, Berlin, Germany); method 2: GRP 3V^®^ module (Scintomics Molecular Applied Theranostics Technologies GmbH, Fürstenfeldbruck, Germany) in conjunction with commercially available SC-01 cassettes (ABX advanced biochemical compounds GmbH, Radeberg, Germany).

**Figure 1:**
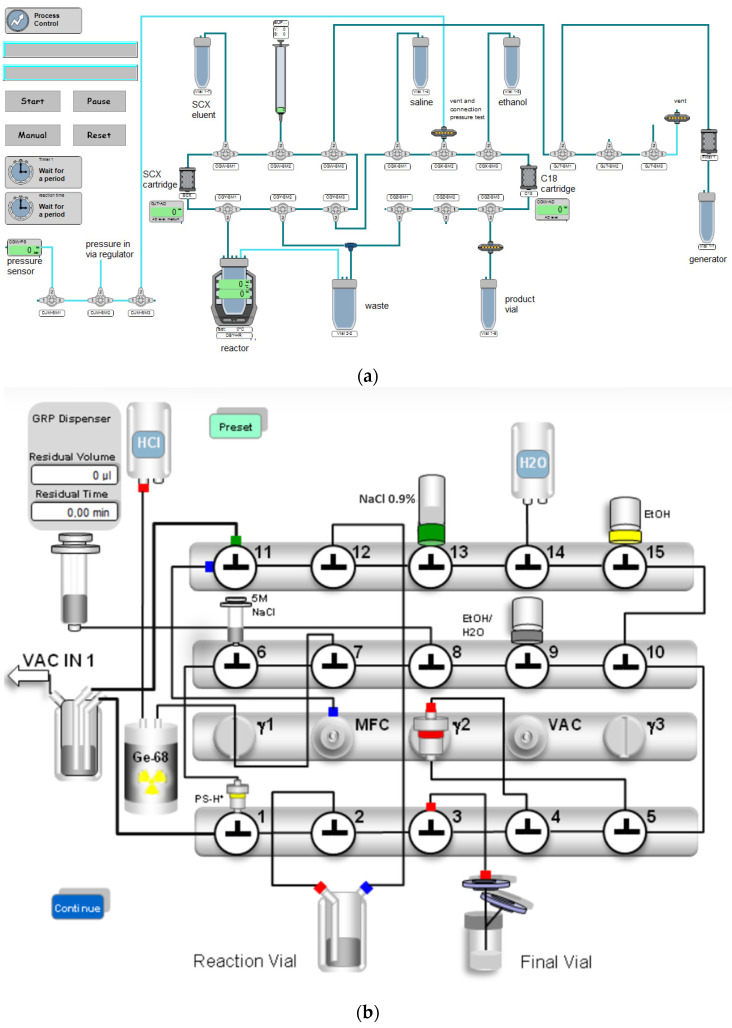
(**a**) Setup for automated synthesis of [^68^Ga]Ga-desferrioxamine B on the Modular-Lab PharmTracer synthesis module (method 1). SCX = strong-cation exchange, generator = ^68^Ge/^68^Ga radionuclide generator. (**b**) Setup for automated synthesis of [^68^Ga]Ga-desferrioxamine B on the GRP 3V synthesis module (method 2). PS-H+ = strong-cation exchange cartridge, NaCl 0.9% = physiological saline solution, Ge-68 = ^68^Ge/^68^Ga radionuclide generator.

The synthesis process for both methods consisted of elution of the ^68^Ge/^68^Ga radionuclide generator, incubation at 40 °C, purification on a reversed-phase cartridge (C18), product formulation with 0.9% NaCl solution, filtration using a 0.22 µM sterility filter, and subsequent filter integrity testing. Schematic representations of the production process for both automated synthesis modules are shown in Figure 1a and Figure 1b, respectively. A more detailed description of both synthesis processes is available in the Supplementary Material.

[^68^Ga]GaCl3 used for radiolabelling was obtained from a licensed GalliaPharm^® 68^Ge/^68^Ga radionuclide generator (1850 MBq theoretical activity) by elution with 9 mL of 0.1 M hydrochloric acid (Ultrapure) (both Eckert & Ziegler Radiopharma GmbH, Berlin, Germany).

Desferrioxamine B was obtained as desferrioxamine B mesylate from a licensed pharmaceutical (Desferal^®^ vials 500 mg, Novartis Pharmaceuticals, Vienna, Austria). One vial was dissolved in 5 mL water for injection, and 100 µL of this solution was diluted to 10 mL with water for injection. A total of 100 µL of this dilution (corresponding to 100 µg desferrioxamine B mesylate) was further diluted with 1.5 mL acetic acid/sodium acetate buffer 1.28 M with pH 4.5 containing 3 mg ascorbic acid. This mixture was used for radiolabelling. The eluate of the ^68^Ge/^68^Ga generator was absorbed on a strong cation exchange cartridge, and [^68^Ga]GaCl_3_ was desorbed with a 4.9 M NaCl/0.14 M HCl solution (method 1) or 5 M sodium chloride solution (method 2) as eluent solution to the buffer containing desferrioxamine B; the reaction solution was heated to 40 °C for 5 min.

Solid phase extraction (SPE) purification of the crude product was conducted via a C18 Sep-Pak^®^ Plus Light cartridge (Waters Corporation, Milford, MA, USA). Physiological saline (method 1) or water for injection (method 2) was used to transfer the labelling solution to the cartridge, wash the reaction vial, and perform all the washing steps. Elution of the purified product from the cartridge was carried out with 50% (*v*/*v*) ethanol (1 mL for method 1; 2 mL for method 2) and the solution directly sterile filtered (0.22 µm filter (Millex-GV filter, 33 mm, Merck KGaA, Darmstadt, Germany)) into the final product vial. This bulk solution was immediately diluted with physiological saline to a final volume of 8.5 mL (method 1) or 17 mL (method 2). The employed sterile filter was tested for filter integrity immediately after the end of synthesis.

[^nat^Ga]Ga-desferrioxamine B as an inactive reference compound was prepared by reacting 100 µg of desferrioxamine B mesylate with 12 µL of GaBr_3_ solution (5 mg/mL in 0.1 N HCl) and 10 µL of sodium acetate trihydrate (155 mg in 1 mL water) at 40 °C for 5 min. Purification was performed on a reversed-phase cartridge (Sep-Pak C18 Plus Light, Waters Corporation, Milford, MA, USA). The cartridge was subsequently washed with water and eluted with ethanol 50%. The resulting purified solution of [^nat^Ga]Ga-desferrioxamine B was stored at −20 °C. The purity of [^nat^Ga]Ga-desferrioxamine B was assessed using RP-HPLC analysis. All chemicals used for synthesis and purification of [^nat^Ga]Ga-desferrioxamine B were reagent grade (Merck, Darmstadt, Germany) apart from desferrioxamine B mesylate, which was available from Desferal^®^. Reference standards of desferrioxamine B mesylate and deferoxamine for system suitability were obtained as Chemical Reference Standard (CRS, EDQM; Strasbourg, France).

### 2.2. Quality Control

Quality control was performed directly after end of synthesis. Stability of the final product was assessed up to 4 h post production.

Instant thin-layer chromatography (ITLC) was performed on silica gel as stationary phase (ITLC-SG, Agilent Technologies, Vienna, Austria), using mobile phases as follows: (A) sodium citrate 0.1 M (pH = 5.0), (B) ammonium acetate 1 M/methanol (1:1 *v*/*v*). Scan-RAM radio-TLC scanner (LabLogic Systems Ltd., Sheffield, UK) with LAURA radiochromatography analysis software (V6.0.2.56) was used for analysis and quantification.

For reversed-phase high-performance liquid chromatography (RP-HPLC) analysis, an UltiMate 3000 system (Thermo Fisher Scientific, Vienna, Austria) was employed with the following components: RS UHPLC pump, autosampler, column compartment (oven temperature 25 °C), variable Wavelength Detector with UV detection at λ = 220 nm. HPLC-radiodetection was achieved by using aGabi Star radiodetector (Raytest; Straubenhardt, Germany) connected in series.

As stationary phase an ACE 3 C18 150 × 3.0 mm column (ACE; Avantor, Radnor, PA, USA) was used, as mobile phases with a flow rate of 0.6 mL/min 0.1% TFA in water (A) and 0.1% TFA in acetonitrile (B) employing the following gradient: 8–18% B (0–12 min); 50% B (12.1–15.0 min); 8% B (15.1–20 min). Calibration for desferrioxamine B quantification was performed based on a calibration curve generated by injection of varying amounts of deferoxamine mesylate CRS using a matrix equivalent to the product formulation (6% ethanol in physiological saline).

pH measurement of buffer solutions and product solutions after radioactive decay was conducted with an Orion 3-Star Benchtop pH meter (Thermo Fisher Scientific, Vienna, Austria). The pH of the product at the end of synthesis was tested using indicator strips (1.09526.0003, Merck KGaA, Darmstadt, Germany).

The determination of ethanol content was performed on a GC-2010 Plus gas chromatograph with an AOC-20i autosampler (both Shimadzu Austria, Korneuburg, Austria), equipped with a Phenomenex Zebron ZB-624 column (7KM-G005-31). A flow rate of 3 mL/min and a FID temperature of 260 °C were applied.

The majority of tests were intended to be performed on each batch before release. Exceptions are justified in the Supplementary Materials.

### 2.3. Process Validation

Four masterbatches of [^68^Ga]Ga-desferrioxamine B for each synthesis module were produced and analysed for validation of the described synthesis approaches. Product specifications were defined based on published Ph. Eur. monographs for [^68^Ga]Ga-PSMA-11 and [^68^Ga]Ga-edotreotide.

## 3. Results

### 3.1. Generator Elution and Reaction Conditions

With the described buffer system, a pH of 4.5 was reached in the reaction solution. Elution of the ^68^Ge/^68^Ga radionuclide generator was followed by pre-concentration of the [^68^Ga]GaCl_3_ eluate using an SCX column. This process resulted in activity yields of 405.9 ± 26.3 MBq for method 1 and 431.6 ± 150.3 MBq for method 2 (both at EOS), with final radioactivity concentrations of 47.8 ± 3.1 MBq/mL (method 1) and 25.4 ± 8.8 MBq/mL (method 2). Molar activities were calculated to be 3.5 ± 0.3 GBq/µmol for method 1 and 4.0 ± 1.4 GBq/µmol for method 2. Radiochemical yields based on the theoretical germanium-68 activity at the time of elution were 64.53 ± 6.09% (method 1) and 49.61 ± 11.27% (method 2). All values for both methods were determined with four individual batches each.

To not exceed the threshold of 10% (*v*/*v*) ethanol according to Ph. Eur. in the final product, the bulk solution was diluted with physiological saline to a final volume of 8.5 mL (method 1) or 17.0 mL (method 2). Ethanol concentration, as determined using gas chromatography analysis of the product formulations, resulted in a value of 6.3 ± 0.2% (*v*/*v*) for method 1 and 5.4 ± 0.4% (*v*/*v*) for method 2.

### 3.2. ITLC & RP-HPLC Analysis and Stability Testing

Product formulations were analysed via instant thin-layer chromatography (ITLC) using mobile phases A and B, which demonstrated radionuclide incorporation of >97%. These values remained similar during stability testing for up to 2 h after the end of synthesis (n = 4). ITLC with sodium citrate 0.1 M (A) was only carried out as part of process validation. ITLC with ammonium acetate 1 M and methanol 1:1 *v*/*v* (B) was defined as a release parameter in analogy to Ph. Eur. monographs [18,19]. Samples of chromatograms for ITLC are shown in the Supplementary Material (Figure S1).

**Figure 2:**
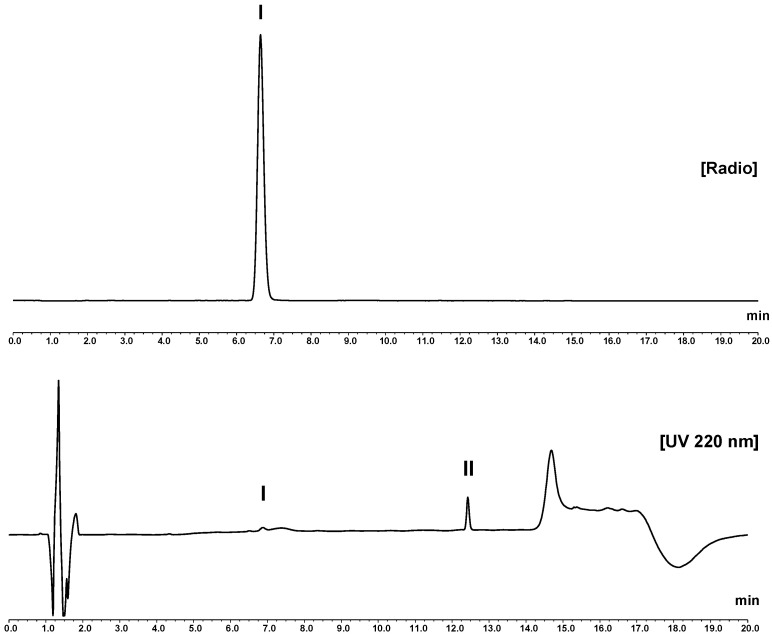
RP-HPLC analysis of [^68^Ga]Ga-desferrioxamine B with the described water/acetonitrile/TFA gradient (column: ACE 3 C18). **Upper chromatogram**: radio-trace. **Lower chromatogram**: UV trace (220 nm). In the radiochromatogram, the principle peak (I) at 6.6 min shows a similar retention to the UV peak of reference compound [^nat^Ga]Ga-desferrioxamine B (see Figure S2 provided in the Supplementary Material). The main peak of unlabelled desferrioxamine B (II), with a retention time of 12.4 min, matches the peak (UV) of desferrioxamine B from Desferal^®^ and deferoxamine CRS standard for system suitability (see Figures S3 and S4 in the Supplementary Material).

A main peak in the radiochromatogram at approximately 6.6 min (see Figure 2) was detected during RP-HPLC analysis, matching the retention time of the main peak in the UV chromatogram obtained for the reference compound [^nat^Ga]Ga-desferrioxamine B (see Figure S2). The method provided an excellent separation from desferrioxamine B itself (range of t_R_ = 11.9–12.5 min, see Figures S3 and S4) with a resolution of >20 and also separation of the two peaks in the deferoxamine for system suitability chemical reference standard (CRS) (Figure S4) with a resolution of >3 for the impurity meeting the specifications in the Ph. Eur. monograph for deferoxamine mesilate [20].

HPLC analysis conducted immediately after production showed a mean radiochemical purity of 98.2 ± 0.5% for method 1 and 98.6 ± 1.3% for method 2 (n = 4). Two- and four-hour post-production stability testing, which verified the high stability of the product formulation resulting in an RCP of 98.0 ± 0.6% (2 h) and 97.6 ± 0.5% (4 h) (n = 4 for both), was conducted. By integrating all visible peaks in the UV trace of the chromatogram, precursor concentration in the product solution was established. Subsequently, these peak areas were compared to peak areas of a calibration curve of desferrioxamine B, giving values of 103.7 ± 4.8 µg/V for method 1 and 96.9 ± 5.2 µg/V for method 2 (n = 4 for both; see Table 1).

### 3.3. Process Validation

Specifications of Ph. Eur. monographs of other ^68^Ga-labelled radiopharmaceuticals [18,19] and findings from radiolabelling pre-tests were employed to define quality criteria for clinical preparation. For process validation four masterbatches of both production routes were prepared. All of these were in compliance with the pre-defined quality criteria and specifications (see Table 1). More in-depth justification of specifications can be found in the Supplementary Material. 

## 4. Discussion

[^68^Ga]Ga-desferrioxamine B showed highly promising results in preclinical studies [11], with a high sensitivity to detect infections with several clinically relevant bacteria, including *Staphylococcus* or *Pseudomonas* ssp. Contrary to currently available PET radiotracers for imaging of infectious processes, in particular [^18^F]FDG, which only visualises the effects of these pathogens, i.e., inflammatory response, [^68^Ga]Ga-desferrioxamine B can potentially provide imaging data of the actual causative microorganism in vivo and would potentially allow to monitor the origin of infections in patients, as well as treatment response. The short half-life of Ga-68 is suitable for this application, as the radiation burden for patients can be kept relatively low compared with other radionuclides, which would be suitable for the radiolabelling of desferrioxamine B, e.g., Ga-67 or Zr-89 [21,22], but in particular, based on its availability from a radionuclide generator, allows for the immediate and on-demand preparation of the PET radiopharmaceutical. Even though also other siderophores have been proposed, such as [^68^Ga]Ga-pyoverdine, which showed high specific uptake of this radiotracer in *Pseudomonas aeruginosa* [23], [^68^Ga]Ga-desferrioxamine B was selected for a first in human clinical trial due to the availability of desferrioxamine B as a licensed pharmaceutical for human use.

As previously shown [11], the radiolabelling of desferrioxamine B with Ga-68 can be performed under mild conditions and yields a product of high radiochemical purity. For a clinical translation, automation of the process was required to allow for the preparation of radiopharmaceuticals according to current radiopharmaceutical standards and to reduce the radiation burden on operators. The alternative of providing a kit formulation, as established for, e.g., [^68^Ga]Ga-DOTA-TOC or [^68^Ga]Ga-PSMA-11, was discarded due to the additional efforts for a GMP-compliant kit production [24]. The synthesis was established on two different synthesis modules to enable the inclusion of different centres into the planned clinical trial. Setup of the automated production process based on single-use cassette systems was established in alignment with well-known procedures for ^68^Ga-radiolabelling [25] and based on the preclinical data on [^68^Ga]Ga-desferrioxamine B preparation [11]. Pre-concentration of [^68^Ga]GaCl_3_ was employed to keep the reaction volume low and to compensate for differences between generator batches, which might result in variable elution volume and, therefore, pH variance during radiolabelling. An acetate buffer system with pH 4.5, higher than for ^68^Ga-labelling of DOTA-based radiopharmaceuticals [26], with ascorbic acid as a radical scavenger, was used. 100 µg desferrioxamine B mesylate precursor was used to enable a stable labelling process while guaranteeing sufficiently low amounts to ensure the tracer principle. Since desferrioxamine B is given in much higher amounts of up to 50 mg/kg body weight for treatment of iron overload, this limit was considered safe for patient use [27]. The complex formation of Ga-68 with desferrioxamine B can be achieved at ambient temperature; for automated synthesis, mild heating at 40 °C was employed to ensure sufficient and stable product yields. The batch results reported here confirm high yields and high radiochemical purity of the final product with the described synthesis approaches. One major difference between the two employed synthesis modules was that for Method 2, a higher final volume was required, as the applied synthesis setup requires larger ethanol amounts to elute the purified product from the C18 cartridge. Therefore, twice as much saline was required to not keep the products below the pharmacopeial limit of 10% (*v*/*v*) ethanol [18].

Overall, these conditions resulted in products of equivalent quality with high radiochemical purity on both synthesis modules and meeting quality specifications analogous to well-established ^68^Ga-radiopharmaceuticals. For HPLC analysis, reference standards from Ph. Eur. were available, and the applied method resulted in excellent resolution between desferrioxamine B and its respective gallium-complex, which was also used to establish a method for system suitability testing and also sufficient separation of the impurity in the system suitability standard CRS from Ph. Eur. 

HPLC analysis also allowed to determine the content of desferrioxamine B in the final product formulations by integrating the corresponding UV-peaks following comparison with a calibration curve. To include variations from precursor dilutions the specifications for the validation procedures were set to 150 µg desferrioxamine B per V. 

Product stability was confirmed over 2 h post-production through radiochemical purity analysis via RP-HPLC and ITLC. Moreover, 4 h measurements were performed but were inconclusive owing to low detectability and low signal-to-noise ratio. Consequently, a shelf life of the final products of 2 h was defined.

This study was the basis for the initiation of an academic clinical trial, which is currently ongoing at the Medical University of Innsbruck under the EudraCT-No. 2020-002868-31 titled “A Phase I/IIa study to evaluate the safety, biodistribution, dosimetry and preliminary diagnostic performance of [^68^Ga]Ga-Deferoxamine for PET imaging in patients with bacterial infections”. The data presented herein were the basis of approval of [^68^Ga]Ga-desferrioxamine B as an Investigational Medicinal Product within this trial by the competent Austrian authority. An excerpt of relevant parts of the submitted Investigational Medicinal Product Dossier is available in the Supplementary Material.

## 5. Conclusions

In this paper, we described two approaches for the preparation of [^68^Ga]Ga-desferrioxamine B using two different synthesis modules (Modular-Lab PharmTracer and Scintomics GRP 3V). Both methods resulted in high reproducibility and stable activity yields. All quality control specifications, which were pre-defined based on Ph. Eur. monographs for established ^68^Ga radiopharmaceuticals, were met. This included, in particular, high radiochemical purity as determined through RP-HPLC and ITLC analysis, thus verifying the reliable clinical production of this novel radiopharmaceutical for patient use. These data should also serve other research groups as a valuable reference for the introduction of novel ^68^Ga-radiopharmaceuticals in clinical research.

## Figures and Tables

**Table 1:** Quality control results from four masterbatches (n = 4) for each synthesis module with the defined specifications and methods for [^68^Ga]Ga-desferrioxamine B. All nominal values are given as means ± standard deviation. t_R_ = retention time; t_½_ = half-life; keV = kiloelectronvolt; GC = gas chromatography; EOS = end of synthesis; NMT = not more than (upper limit); NLT = not lower than (lower limit); LoD = limit of detection; V = total volume (8.5 mL for Modular-Lab PharmTracer; 17.0 mL for GRP 3V).

Parameter	Method	Specification	Testing Schedule	Results for Modular-Lab PharmTracer	Results for GRP 3V
Appearance	visual inspection	clear, colourless solution, free from visible particles	prior to release	conforms	conforms
Volume	visual inspection	8.5 ± 1 mL for Modular-Lab PharmTracer//17.0 ± 1 mL for GRP 3V	prior to release	8.5 ± 1 mL	17.0 ± 1 mL
pH	pH indicator strip	4–8	prior to release	5.4 ± 0.2	5.0 ± 0.1
Identity HPLC	RP-HPLC	according to [^nat^Ga]Ga-desferrioxamine B standard (relative t_R_ 0.9–1.1)	prior to release	conforms	conforms
Activity yield	dose calibrator	NLT 200 MBq (at EOS)	prior to release	405.9 ± 26.3 MBq	431.6 ± 150.3 MBq
Radioactivity concentration	dose calibrator	20–150 MBq/mL for Modular-Lab PharmTracer//10–75 MBq/mL for GRP 3V	prior to release	47.8 ± 3.1 MBq/mL	25.4 ± 8.8 MBq/mL
Radionuclidic identity (t_½_)	dose calibrator	68 ± 6 min	during qualification of generator	68 min	68 min
Radionuclidic identity	gamma-ray spectrometry	γ line at 511 keV; γ line at 1022 keV; γ line at 1077 keV (optional)	during qualification of generator	conforms	conforms
Chemical purity and content	RP-HPLC	desferrioxamine B, Ga-desferrioxamine B and related impurities NMT 150 µg/V	prior to release	103.7 ± 4.8 µg/V	96.9 ± 5.2 µg/V
	GC	Ethanol NMT 10% (*v*/*v*)	after release	6.3 ± 0.2%	5.4 ± 0.4%
Radiochemical purity	RP-HPLC	[^68^Ga]Ga-desferrioxamine B: NLT 95%	prior to release	98.2 ± 0.5%	98.6 ± 1.3%
	TLC (ammonium acetate 1 M + methanol 1:1)	colloidal [^68^Ga]Ga: NMT 3%	prior to release	0.5 ± 0.3%	1.2 ± 1.4%
Radionuclidic purity	gamma ray spectrometry	^68^Ge: NMT 0.001% (after ≥48 h)	after release	conforms (below LoD)	conforms (below LoD)
Filter integrity	pressure hold test	based on programme of synthesis module	prior to release	conforms	conforms

## Data Availability

Data are contained within the article and Supplementary Materials.

## Supplementary Materials

The following supporting information can be downloaded at https://www.mdpi.com/article/10.3390/pharmaceutics16091231/s1, Figure S1: Sample chromatograms from ITLC of [^68^Ga]Ga-desferrioxamine B with silica gel as stationary phase; Figure S2: Sample UV chromatogram at 220 nm from RP-HPLC of [^nat^Ga]Ga-desferrioxamine B; Figure S3: Sample UV chromatogram at 220 nm from RP-HPLC of desferrioxamine B (Desferal^®^); Figure S4: Sample UV chromatogram at 220 nm from RP-HPLC of desferrioxamine B (CRS standard for system suitability). Figure S5a: Detailed flowchart of production of [^68^Ga]Ga-desferrioxamine B on Modular-Lab Pharmtracer; Figure S5b: Detailed flowchart of production of [^68^Ga]Ga-desferrioxamine B on GRP 3V [28].
