# Supplementary material for: Automated Production of [68Ga]Ga-Desferrioxamine B on Two Different Synthesis Platforms
Source: Pharmaceutics. 2024 Sep 21;16(9):1231. doi: 10.3390/pharmaceutics16091231 (PMC11435116; doi:10.3390/pharmaceutics16091231)
Supplement: Supplementary file 1 [file pharmaceutics-16-01231-s001.zip › pharmaceutics-3146294-supplementary.pdf]

Supplementary material

A- TLC and HPLC chromatograms

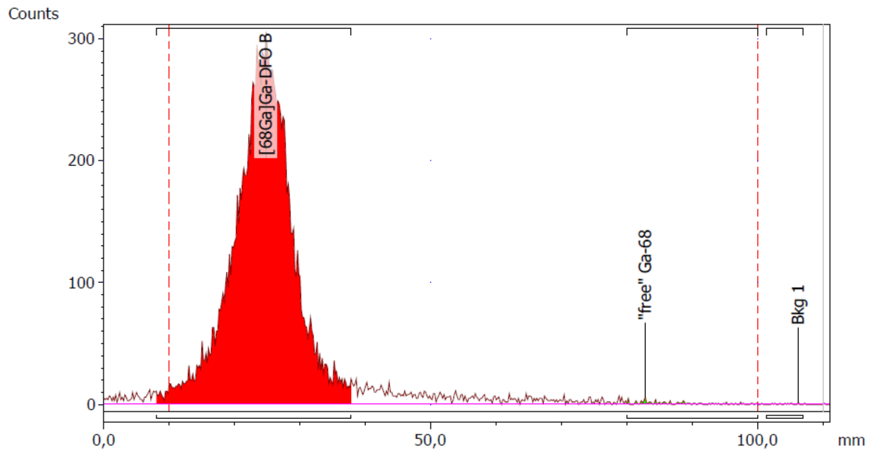

Solvents: 0,1 M NaCitrat pH5
Regions: ^{68}Ga_GaDFOB200507cd Run 1

Name	Start (mm)	End (mm)	Centroid (mm)	Retention (RF)	Area (Counts)	%ROI (%)	%Total (%)
[68Ga]Ga-DFO B	8,0	37,8	24,4	0,165	14663	99,64	90,97
"free" Ga-68	80,0	100,0	84,4	0,809	54	0,36	0,33
Bkg 1	101,4	107,0	101,4	1,069			
2 Peaks					14717	100,00	91,30

Total Area: 16120 Counts
Average Background: 0 Counts

Method: Ga68_v1_l100

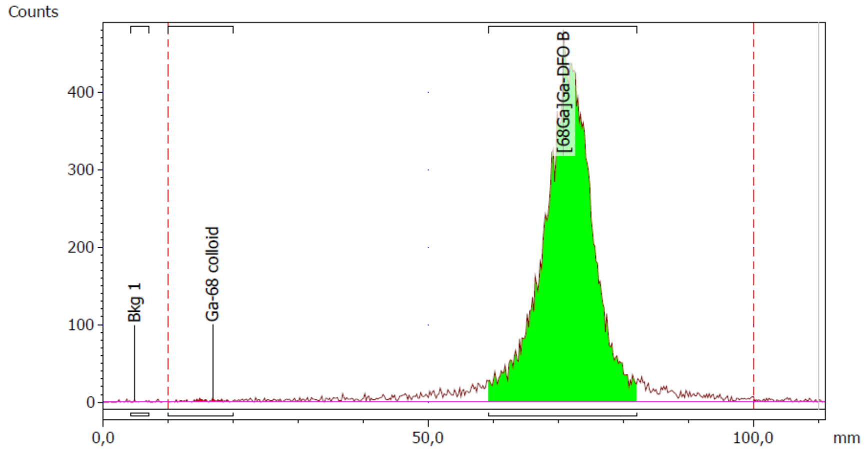

Solvents: MeOH/NH4 Acetat (1/1)
Regions: ^{68}Ga_GaDFOB200507cd Run 2

Name	Start (mm)	End (mm)	Centroid (mm)	Retention (RF)	Area (Counts)	%ROI (%)	%Total (%)
Bkg 1	4,2	7,0	4,2	-0,058			
Ga-68 colloid	10,0	20,0	15,6	0,076	47	0,25	0,22
[68Ga]Ga-DFO B	59,2	82,0	71,4	0,676	19094	99,75	89,91
2 Peaks					19141	100,00	90,13

Total Area: 21237 Counts
Average Background: 1 Counts

Method: Ga68_v1_l100

Figure S1 Sample chromatograms from ITLC of [^{68}Ga]Ga-desferrioxamine B with silica gel as stationary phase. Chromatograms with mobile phase A (**upper panel**: sodium citrate 0.1 M with pH 5) and mobile phase B (**lower panel**: ammonium acetate 1 M + methanol 1:1 $^{v}/_{v}$) are shown.

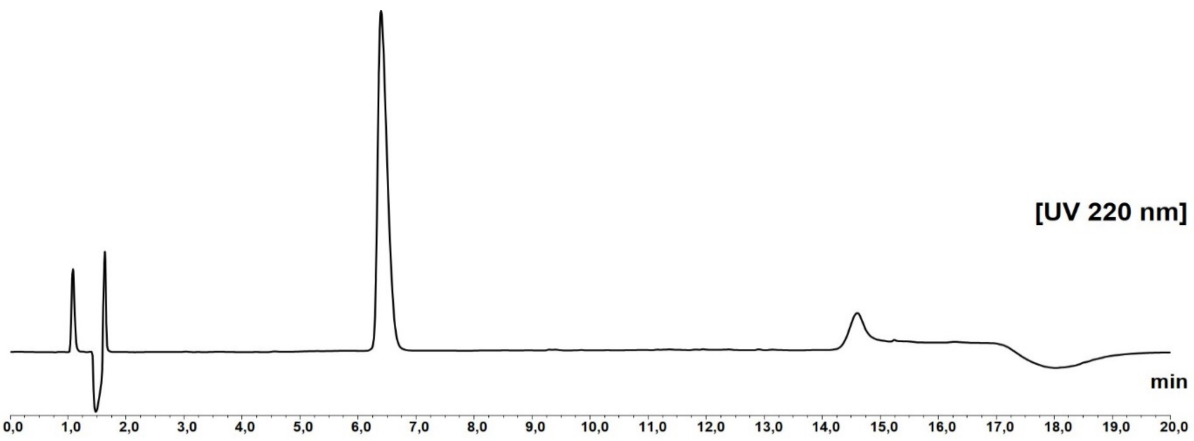

Figure S2 Sample UV chromatogram at 220 nm from RP-HPLC of [^{nat}Ga]Ga-desferrioxamine B. The main peak has a retention time of 6.4 min.

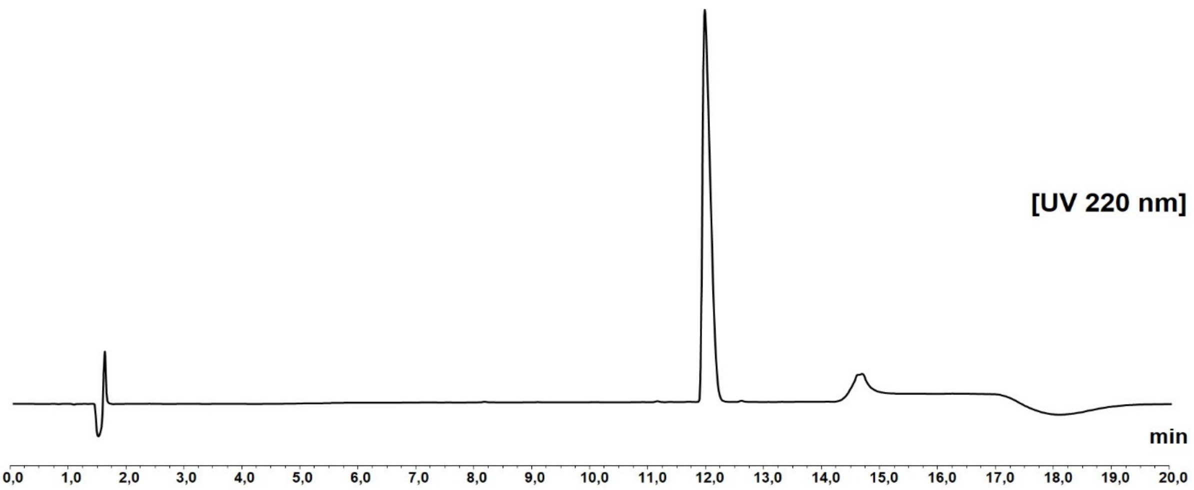

Figure S3 Sample UV chromatogram at 220 nm from RP-HPLC of desferrioxamine B (Desferal®). The main peak has a retention time of 11.9 min.

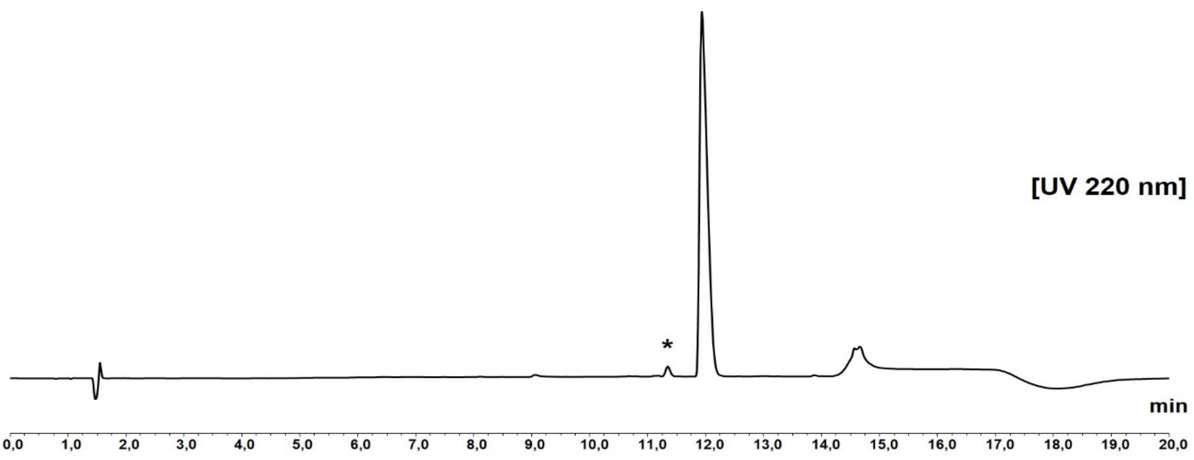

Figure S4 Sample UV chromatogram at 220 nm from RP-HPLC of desferrioxamine B (CRS standard for system suitability). The main peak has a retention time of 11.9 min. An impurity specified as impurity G in the Ph. Eur. is visible at 11.3 min (marked with an asterisk * in the chromatogram).

B- Excerpt from the Investigational Medicinal Product Dossier (IMPD)

The nomenclature and numbering of the following text is based on the EMA document "Guideline on the requirements to the chemical and pharmaceutical quality documentation concerning investigational medicinal products in clinical trials".

2.1.S.2.2.2 Description of the Manufacturing Process and Process Control

2.1.S.2.2.2.c Automated Manufacturing Process

Manufacturing is performed using one of the following automated radiosynthesis modules:

- Modular-Lab Pharmtracer manufactured by Eckert & Ziegler Eurotope GmbH.
- GRP 3V manufactured by SCINTOMICS Molecular Applied Theranostics Technologies GmbH (ATT).

The principle process of the two modules is almost identical. The automated manufacturing process is described in detail for each module below. Differences between the two automated manufacturing processes are described at the end of this chapter.

Before the automated synthesis starts, the reaction vial is pre-loaded with precursor material Desferoxamine mesilate (100 µg in 100 µL water) diluted with 1.5 mL of sodium acetate buffer prepared from sodium acetate, acetic acid and water with addition of ascorbic acid.

Narrative description for Modular-Lab Pharmtracer:

Sep-Pak® Light C18 cartridge is pre-conditioned using ethanol and saline.

$[^{68}Ga]GaCl_3$ is eluted from the generator with diluted hydrochloric acid. The solution is passed through a cation exchange cartridge (type Eichrom SCX-Silica). The material on the cartridge binds Ga-68, whereas impurities like Ge-68 and other metal cations are not bound and remain in the solution. Ga-68 is then eluted from the SCX-cartridge into the reaction vial using eluent solution. For radiolabelling the reaction mixture is heated to 40°C for 5 min.

For purification, the reaction mixture is passed through a Sep-Pak® Light C18 cartridge and the retained crude $[^{68}Ga]$Ga-desferrioxamine B is washed with saline. $[^{68}Ga]$Ga- desferrioxamine B is extracted (from the Sep-Pak® Light C18 cartridge) using Ethanol/Water (1:1). The Drug Substance $[^{68}Ga]$Ga- desferrioxamine B is transferred into the bulk vial through a sterile filter and formulated with saline.

Remark: The reaction of the radionuclide and the precursor material followed by purification steps is considered the radiosynthesis. Elution of Drug Substance from the purification cartridge and transfer to the bulk vial through a sterile filter followed by adding saline is considered the formulation.

Step-by-step description for Modular-Lab Pharmtracer:

Steps 1-12 represent the radiosynthesis, Steps 13 and 14 represent the formulation:

1. Pre-conditioning of the Sep-Pak® Light C18 cartridge with ethanol/water (1:1) and saline. The filtrate is transferred to the waste.

2. Elution of the $^{68}Ge/^{68}Ga$ radionuclide generator with 0.1 M hydrochloric acid and collection of 9 mL of eluate in the dispenser syringe.

3. Dispensing of this mixture onto the cation exchange cartridge (type SCX-Silica Eichrom), $[^{68}Ga]Ga^{3+}$ is absorbed on the cartridge, the solution is transferred to waste.

4. Drying of the cation exchange cartridge

5. Transfer of eluent solution into dispenser syringe; reactor is pre-heated to 40°C

6. Elution of $[^{68}Ga]Ga^{3+}$ from the cation exchange cartridge using 0.8 mL eluent solution; transfer to reactor vial

7. Labelling reaction for 5 min at 40°C

8. Dispenser syringe, cation exchange cartridge and manifolds are rinsed with saline rinsing solution then transferred to waste

9. Cooling of the reactor

10. Transfer of the reaction mixture to the dispenser syringe

11. Transfer of the reaction mixture to Sep-Pak® Light C18 cartridge; product is absorbed on the cartridge, the solution is transferred to waste

12. Reactor is rinsed using saline, transfer of rinsing solution to Sep-Pak® Light C18 cartridge, then transfer to waste

13. Transfer of 2 mL ethanol/water (1:1) to dispenser syringe; elution of product from the Sep-Pak® Light C18 cartridge with 1mL, transfer into bulk vial through sterile filter, residual ethanol is washed to the waste with saline.

14. Dilution of the final product solution with 7.5 mL of saline, added through sterile filter

Final volume: 8.5 mL ± 1 mL

Total synthesis time: approximately 35 min

Batch size [Radioactivity concentration (MBq/mL) at EOS]: 20 – 150 MBq/mL

The detailed synthesis process using the Modular-Lab Pharmtracer module is depicted in Figure S5a below.

Narrative description for GRP 3V:

Sep-Pak® Light C18 cartridge is pre-conditioned using ethanol and water.

$[^{68}Ga]GaCl_3$ is eluted from the generator with diluted hydrochloric acid. The solution is passed through a cation exchange cartridge (type SCX PS-H^+). The material on the cartridge binds Ga-68, whereas impurities like Ge-68 and other metal cations are not bound and remain in the solution. Ga-68 is then eluted from the SCX-cartridge into the reaction vial using eluent solution. For radiolabelling the reaction mixture is heated to 40°C for 5 min.

For purification, the reaction mixture is passed through a Sep-Pak® Light C18 cartridge and the retained crude $[^{68}Ga]$Ga-desferrioxamine B is washed with water. $[^{68}Ga]$Ga-desferrioxamine B is extracted (from the Sep-Pak® Light C18 cartridge) using Ethanol/Water (1:1). The Drug Substance $[^{68}Ga]$Ga-desferrioxamine B is transferred into the bulk vial through a sterile filter and formulated with saline.

Remark: The reaction of the radionuclide and the precursor material followed by purification steps is considered the radiosynthesis. Elution of Drug Substance from the purification cartridge and transfer to the bulk vial through a sterile filter followed by adding saline is considered the formulation.

Step-by-step description for GRP 3V:

Steps 1-11 represent the radiosynthesis, Steps 13 and 14 represent the formulation:

1. Pre-conditioning of the Sep-Pak® Light C18 cartridge with ethanol and water. The filtrate is transferred to the waste.

2. Elution of the $^{68}Ge/^{68}Ga$ radionuclide generator with 0.1 M hydrochloric acid and collection of 9 mL of eluate in the dispenser syringe.

3. Dispensing of this mixture onto the cation exchange cartridge (type SCX PS-H^{+}), $[^{68}Ga]Ga^{3+}$ is absorbed on the cartridge, the solution is transferred to waste.

4. Drying of the cation exchange cartridge

5. Dispenser syringe and manifolds are rinsed with water for injection then transferred to waste

6. Transfer of eluent solution into dispenser syringe; reactor is pre-heated to 40°C

7. Elution of $[^{68}Ga]Ga^{3+}$ from the cation exchange cartridge using 1.5 mL eluent solution; transfer to reactor vial

8. Labelling reaction for 5 min at 40°C

9. Cooling of the reactor

10. Transfer of the reaction mixture to the dispenser syringe

11. Transfer of the reaction mixture to Sep-Pak® Light C18 cartridge; product is absorbed on the cartridge, the solution is transferred to waste

12. Reactor is rinsed using water, transfer of rinsing solution to Sep-Pak® Light C18 cartridge, then transfer to waste

13. Transfer of 2 mL ethanol/water (1:1) to dispenser syringe; elution of product from the Sep-Pak® Light C18 cartridge with 2 mL, transfer into bulk vial through sterile filter

14. Dilution of the final product solution with 15 mL of saline, added through sterile filter

Final volume: 17 mL ± 1 mL

Total synthesis time: approximately 35 min

Batch size [Radioactivity concentration (MBq/mL) at EOS]: 10 – 75 MBq/mL

The detailed synthesis process using the GRP 3V module is depicted in Figure S5b below.

A major difference between the two automated manufacturing processes is the final product volume (8.5 mL for Modular-Lab Pharmtracer as compared to 17 mL for GRP 3V). The discrepancy in final volume is a result of different dilution steps performed at the end of the automated manufacturing process, resulting in different radioactivity concentrations at EOS and therefore different minimum and maximum batch sizes, as outlined above. The difference in final volume for these two processes also results in different container closure systems.

Other minor differences include the use of a different SCX cartridge and usage of water for injection instead of saline for rinsing and washing steps on the GRP 3V module. However, since none of these

steps influence the final product formulation, the resulting radiopharmaceutical is considering equivalent in quality with only the final volume (as described above) varying between the two production processes.

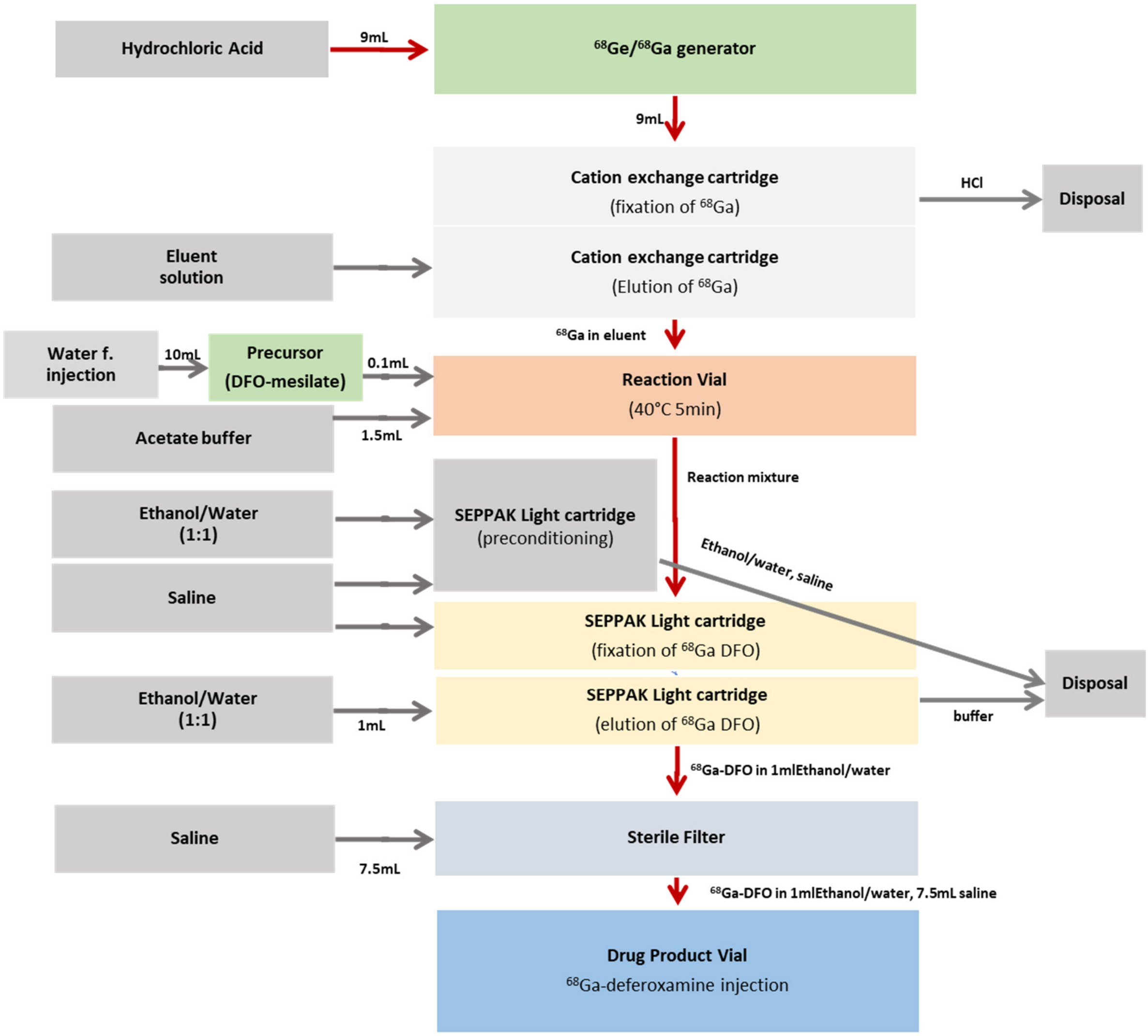

Figure S5a: Detailed flowchart of production of [^{68}Ga]Ga-desferrioxamine B on Modular-Lab Pharmtracer

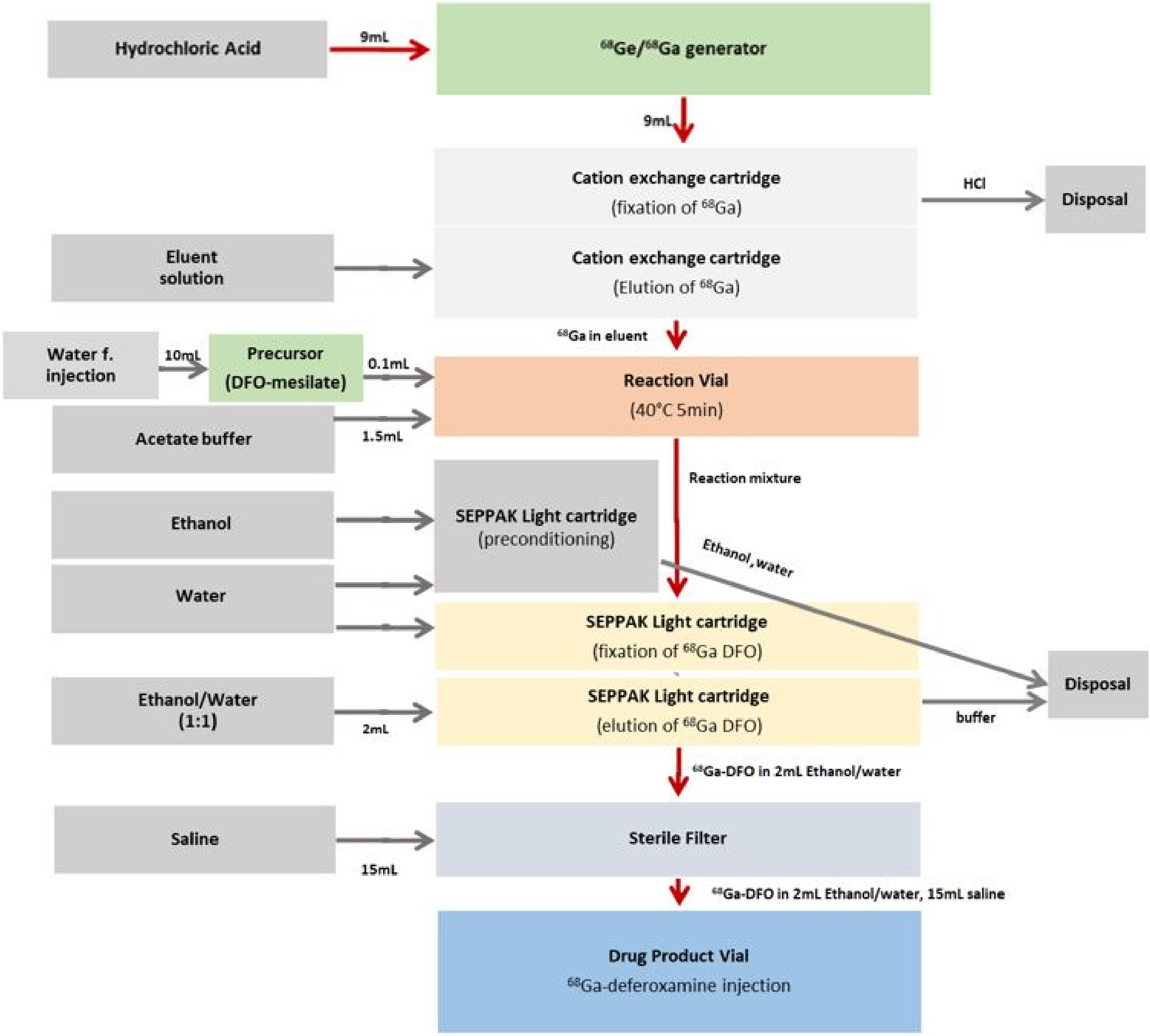

Figure S5b: Detailed flowchart of production of [^{68}Ga]Ga-desferrioxamine B on GRP 3V

2.1.P.5.6 Justification of specification(s)

The following relevant Ph. Eur. monographs have been considered for establishing the Drug Product specification:

- Radiopharmaceutical preparations (Ph. Eur. 125)
- Table of physical characteristics of radionuclides (Ph. Eur. 5.7)
- Gallium (^{68}Ga) Edotreotide injection (Ph. Eur. 2482)
- Gallium (^{68}Ga) Chloride Solution for Radiolabelling (Ph. Eur. 2464)
- Fluoroethyl-L-Thyrosine (F-18) injection (Ph. Eur. No 2466)
- Gallium (^{68}Ga) PSMA-11 Injection (Ph. Eur. 3044)
- Deferoxamine Mesilate (Ph. Eur. 0896)

The majority of tests are performed on each batch before release. Exceptions are justified below.

2.1.P.5.6.1 Maximum volume of one patient dose and radioactivity concentration

The maximum volume to be injected is 5 mL for the drug product produced with Modular-Lab PharmTracer and 10 mL produced with GRP 3V. This takes into account the required sampling volumes for quality control and is reflected in the activity concentration specification with a range of 20-150 MBq/mL (Modul-Lab PharmTracer) and 10-75 MBq/mL (GRP 3V), respectively. The lower limit is based on the minimum activity required for a patient dose of 100 MBq, which is 20 MBq/mL with a max volume of 5 mL for the drug product produced with Modular-Lab PharmTracer and 10 MBq/mL with a max volume of 10 mL for the drug product produced with GRP 3V.

The upper radioactivity concentration limit of 150 MBq/mL (Modular-Lab PharmTracer) and 75 MBq/mL (GRP 3V) is based on the maximum activity of a ^{68}Ge/^{68}Ga radionuclide generator and the yields of production that cannot exceed 1250 MBq in total. Even though the maximum volume to be injected in a patient (V) is defined as 5 mL (Modul-Lab PharmTracer) or 10 mL (GRP 3V), for safety reasons all limits related to a volume V are considered for the total volume of 8.5 mL (Modul-Lab PharmTracer) or 17 mL (GRP 3V).

The difference in maximum volume of one patient dose is a direct result of dissimilar dilution steps at the end of the two automated synthesis processes.

2.1.P.5.6.2 Radionuclidic Identity of Ga-68

The radionuclidic identity of Ga-68 is confirmed by half-life determination and by gamma ray spectroscopy by evaluating the nature and energy of the radiation as described in the Ph. Eur. monograph "Radiopharmaceutical Preparations". The half-life and radiation of Ga-68 is also described in the Ph. Eur. [Table of physical characteristics of radionuclides (5.7)].

2.1.P.5.6.3 Radionuclidic purity

The ^{68}Ge/^{68}Ga radionuclide generator potentially releases Ge-68. The risk for the product to exceed the specified Ge-68 limit is practically eliminated by the measures mentioned above (refer to section 2.1.P.5.5, radioactive by-products).

The limit is adopted from the current Ph. Eur. Gallium (Ga-68) Chloride Solution for Radiolabelling. The test can be done only after decay of the product for at least 48 h. The test will also be conducted regularly on the generator eluate. This is considered safe because rising levels of Ge-68 would be detected early by the regular checks. The generator is used as outlined in the SPC and the production process depletes Ge-68 from the final product. No radionuclides other than Ge-68 or Ga-68 are expected.

2.1.P.5.6.4 Radiochemical purity

There are two types of radiochemical impurities that could be considered:

1. Impurities detected by the HPLC that represent degraded or altered [^{68}Ga]Ga-desferrioxamine B species, presumably formed by radiolytic processes during the radiolabelling and purification. The limit is set to 95% as proposed in the Ph. Eur. monographs for Gallium (^{68}Ga) Edotreotide injection and Gallium (^{68}Ga) PSMA-11 injection. However, never within the development of the process such impurities were detected.
2. Potential free or colloidal Ga-68, not bound to the chelator, which is detected by TLC but cannot be quantitatively detected by the HPLC method (partial retention on HPLC columns). The Ph. Eur. monographs for Gallium (^{68}Ga) Edotreotide injection and Gallium (^{68}Ga) PSMA-11 injection do not specify these impurities anymore, as analytical tests in general cannot reliably distinguish these. The limit is set to NMT 3 %, which is the same limit given in the aforementioned monographs.. In addition to this, purification by use of a C-18 cartridge efficiently eliminates both species.

2.1.P.5.6.5 Limit test of desferrioxamine B and related substances

Desferrioxamine B is used in a licensed formulation in quantities up to more than 5 g/day. Therefore the limit of desferrioxamine B is not based on safety concerns, but to have a control of the used precursor it is set to NMT 150 µg/V. Considering the use of 100 µg precursor this limit ensures to control the process without giving unnecessary constraints in the analytics. This way it is ensured that the maximum amount injected with a max V of 5 mL or 10 mL (see above) is less than 100 µg. Other impurities than desferrioxamine B are potential metal complexes of desferrioxamine B, other related substances are controlled by the use of desferrioxamine B from a licensed product.

The 100 µg limit is also found in other F-18 PET monographs (e.g. Fluoroethyl-L-Thyrosine (F-18) injection Ph. Eur. 2466).

2.1.P.5.6.6 Ethanol

Ethanol is added as an excipient and thus not considered to be a residual solvent. The manufacturing process requires 1 mL of Ethanol 50% (v/v) with the Modular-Lab PharmTracer module or 2 mL of Ethanol 50% (v/v) with the GRP 3V module for complete elution of the Drug Substance from the purification cartridge. This results in a final concentration of NMT 10 % (v/v) in the finished drug product.

Ethanol is frequently used in radiopharmaceutical injections. It can be safely used at a concentration not exceeding 10% and an injection volume below 20 mL [28].

2.1.P.5.6.7 Sterility

As per the general monograph of the Ph. Eur. for Radiopharmaceutical Preparations, radiopharmaceuticals for parenteral application have to be sterile and sterility has to be tested in every batch. Due to the short half-life of Ga-68, [^{68}Ga]Ga-desferrioxamine B injection is released prior to completion of the test.

2.1.P.5.6.8 Bacterial endotoxins

The limit (not more than 175 I.U./maximal applicable dose) is a standard limit derived from Ph. Eur. guidance e.g. the current Ph. Eur. monograph for Fluorodeoxyglucose (F-18) injection and for Gallium (^{68}Ga) Edotreotide injection. The limit was set to 9.5 IU/mL considering a volume of up to 18 mL, depending on the automated synthesis method.

2.1.P.5.6.9 Total activity / range of patient dose (radioactivity)

The limit for the applicable dose range is based on clinical experience, which allows 100-200 MBq Ga-68 activity to acquire high quality PET images.

The 200 MBq will result in an estimated radiation dose of about 3 mSv, which is in the range of most routinely applied PET investigations.

2.1.P.5.6.10 Sterile filter integrity test

The specification of the manufacturer is applied, ensuring proof that the sterile filter integrity is preserved in the process.
